# Monitoring the Aging and Edible Safety of Pork in Postmortem Storage Based on HSI and Wavelet Transform

**DOI:** 10.3390/foods13121903

**Published:** 2024-06-17

**Authors:** Anguo Xie, Yu Zhang, Han Wu, Meng Chen

**Affiliations:** 1 Zhang Zhongjing School of Chinese Medicine, Nanyang Institute of Technology, Nanyang 473000, China; ngsp2101@126.com (H.W.); nyfood24@126.com (M.C.); 2 School of Intelligent Manufacturing, Nanyang Institute of Technology, Nanyang 473000, China; zhangyu@nyist.edu.cn

**Keywords:** postmortem storage, rigor mortis, aging, hyperspectral imaging, wavelet transform, visualizing, pork

## Abstract

The process of meat postmortem aging is a complex one, in which improved tenderness and aroma coincide with negative effects such as water loss and microbial growth. Determining the optimal postmortem storage time for meat is crucial but also challenging. A new visual monitoring technique based on hyperspectral imaging (HSI) has been proposed to monitor pork aging progress. *M. longissimus thoracis* from 15 pigs were stored at 4 °C for 12 days while quality indexes and HSI spectra were measured daily. Based on changes in physical and chemical indicators, 100 out of the 180 pieces of meat were selected and classified into rigor mortis, aged, and spoilt meat. Discrete wavelet transform (DWT) technology was used to improve the accuracy of classification. DWT separated approximate and detailed signals from the spectrum, resulting in a significant increase in classification speed and precision. The support vector machine (SVM) model with 70 band spectra achieved remarkable classification accuracy of 97.06%. The study findings revealed that the aging and microbial spoilage process started at the edges of the meat, with varying rates from one pig to another. Using HSI and visualization techniques, it was possible to evaluate and portray the postmortem aging progress and edible safety of pork during storage. This technology has the potential to aid the meat industry in making informed decisions on the optimal storage and cooking times that would preserve the quality of the meat and ensure its safety for consumption.

## 1. Introduction

The slaughter of animals initiates a series of complex biochemical reactions in the carcass [1]. After an animal dies, its muscles undergo rigor mortis wherein they contract and harden, leading to decreased water retention and poor flavor and edible quality. If meat is stored continuously, proteases present in the meat can degrade cytoskeleton proteins in muscle fibers, rendering them tender and suitable for processing [2]. Furthermore, protein degradation increases the content of free amino acids, and creatine phosphate is decomposed into inosine monophosphate (IMP) during storage, leading to an enhancement in the taste and aroma of meat [3,4]. This phenomenon is known as postmortem aging storage. Proper aging and tenderizing techniques can significantly improve meat tenderness and enhance its overall quality.

However, it is crucial to note that the hardness of meat will not decrease indefinitely [5]. The thickness of muscle fibers and the content of collagen are two factors that significantly impact the final hardness of meat, which is commonly referred to as “background hardness” or “basic hardness”. Additionally, postmortem storage fosters the growth of bacteria that may adversely affect the meat’s shelf-keeping qualities. Extended storage periods can result in water loss and meat dryness, which could potentially reduce the economic benefits for meat producers. Furthermore, microbial contamination and oxidative rancidity of fats can compromise not only the economic interests of meat producers but also the safety of the meat consumers.

Thus, the most desirable meat products are those that are properly aged to achieve the best tenderness, taste, and aroma before spoilage occurs. Determining the optimal postmortem storage time for a piece of meat is a meaningful task. However, accomplishing this goal proves to be quite challenging as the tenderization rate of each meat piece varies based on factors such as age, sex, and slaughter methods [4]. Furthermore, the rate of meat spoilage is also influenced by storage conditions such as temperature and humidity. Currently, meat storage time is determined based on the experience and instincts of workers, without considering individual differences in the carcass. While physical and chemical analysis can yield accurate index values, these methods are time-consuming and inefficient. Recent developments in chemical and biological-based technologies such as mass spectrometry, high-performance liquid chromatography, polymerase chain reaction, and enzyme-linked immunosorbent assay have shown promise in detecting microbial identification and determination, but these methods still require prolonged time to complete [6].

In recent years, the combination of spectroscopy and computer vision technology has facilitated the advancement of hyperspectral imaging (HSI) and has garnered significant attention in various practical applications. Predicting crop growth and yield [7], detecting active ingredient content in drugs, and quality testing of fruits [8], meat [9], and fish products are some of the applications where HSI has proven to be valuable. Among these, HSI research in meat products has been particularly extensive. For instance, HSI has been utilized to predict the intramuscular fat and pH across various red meat species and muscle types [10]. Additionally, researchers have employed NIR-HSI to investigate the degree of lipid oxidation in pork during frozen storage [11], and the quantification of the total viable count (TVC) in pork meat using combined HSI and artificial olfaction techniques has also been explored [12]. Spectral models have enabled researchers to detect other values such as total volatile basic nitrogen (TVB-N) and thiobarbituric-acid-reactive substances (TBARS) [13]. However, most of the predictive models in these studies aim at a single quality indicator, leaving room for investigation of more comprehensive evaluations.

HSI imaging produces a series of optical images with the wavelength of light waves, and its data are three-dimensional. Among them, the two-dimensional image information is presented with high resolution, the third dimension is its wavelength information, and its accuracy reaches the level of 2–3 nm. The amount of data produced by the HSI system poses a significant challenge for data processing, transmission, and calculation [14,15]. To address this issue, data compression techniques are often applied [16,17]. Conventional methods that select several representative wavebands to model risk losing crucial information and may impair predictive performance. Wavelet transform (WT) is an information preprocessing technology developed on the basis of short-time Fourier transform. WT overcomes limitations such as the window size not changing with frequency, providing a “time-frequency” window that adapts to different frequencies. Not only does this highlight certain characteristics of the problem, but it also enables time and space localization of frequency. Therefore, WT is regarded as a significant scientific methodological breakthrough alongside Fourier transform.

WT comes in two forms: continuous wavelet transform (CWT) and discrete wavelet transform (DWT). While CWT can be implemented independently, the signals generated by decomposition contain an excessive amount of redundant information [18]. DWT, on the other hand, only requires a model and some adjustments. The signal can be decomposed into approximation coefficients (cA) and detail coefficients (cD), and further decomposed as necessary. For example, when an input signal is decomposed into five stages, the signal can be decomposed into a series of coefficients (cD1, cD2, cD3, cD4, cD5, and cA5). This process, called decomposition analysis, generates a series of coefficients from which the original signal can be reconstructed using inverse discrete wavelet transform (IDWT) with no loss of information.

Currently, there is a lack of literature reports on utilizing HSI technology to detect meat aging in postmortem storage. Evaluating the progress of meat aging requires a comprehensive judgment based on multiple indicators. Meat undergoes a complex process in postmortem storage, in which improved tenderness and aroma coincide with negative effects such as water loss and microbial growth. In this paper, the physicochemical and spectral changes of pork during the whole process from slaughter to spoilage were studied. HSI technology was used to quantitatively evaluate the tenderizing process and deterioration risk of meat products. Additionally, DWT was utilized to extract feature values from HSI data to improve classification accuracy and speed. The use of new technologies in evaluating meat aging is expected to guide the production of high-quality meat products.

## 2. Materials and Methods

### 2.1. Main Process of the Experiment

**Figure 1:**
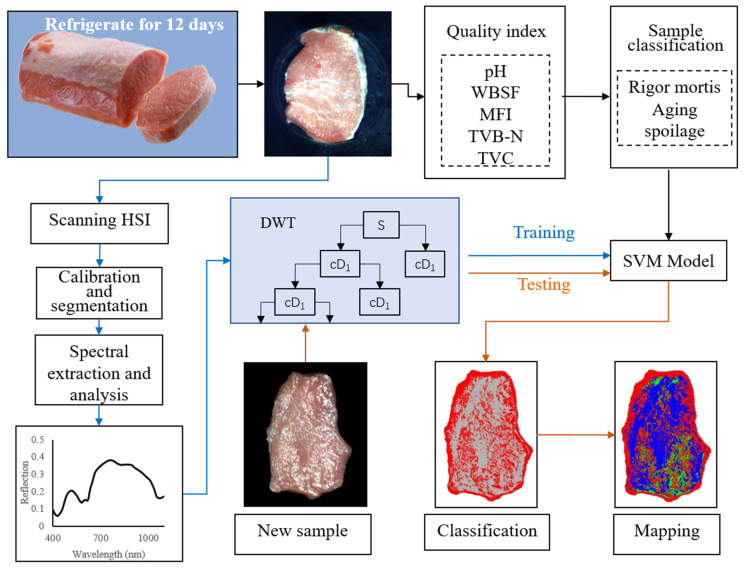
The main steps for HSI to monitor the progress of pork aging.

The main experimental process of meat aging study using HSI technology is shown in Figure 1.

Fifteen porcine specimens were sourced from Muyuan Foods Co., Ltd. (Nanyang, China). Within 20 min postmortem, the *M. longissimus thoracis* was carefully extracted from each specimen by qualified personnel at the facility. These tissue samples were promptly transported under appropriate conditions to our laboratory within a two-hour window. Upon arrival, the meat samples were individually wrapped in food-grade film and stored on shelves in a controlled refrigeration chamber set at 4 ± 0.5 °C with 75% relative humidity for a period of 12 days. Two samples were cut from each pig *M. longissimus thoracis* every day during storage. Each sample has an area of approximately 7 cm × 7 cm, a thickness of 2 cm, and a weight of approximately 100 g. Physical and chemical indexes, including pH, Warner–Bratzler shear force (WBSF), TVB-N, TVC, and myofibrillar fragmentation index (MFI), were measured to determine rigor mortis, aging, and spoilage. To obtain spectral information of the meat, a hyperspectral imaging system (Model IST50-3810, Inno-spec GMBH, Obersulm, Germany) was used to scan the samples. This HSI system contains 512 bands in the 308–1105 nm spectral range, with a spectral resolution of about 2.8 nm. CCD camera pixel is 1004 × 1002, resolution point radius < 9 μm. Spectral analysis was performed using wavelet transform and support vector machine (SVM). In addition, the postmortem aging process of pork was visualized in pseudo color mode.

### 2.2. Determination of Physical and Chemical Indicators of Pork in Cold Storage

#### 2.2.1. pH Values

The pH of the samples was measured using a pH meter (Testo 205, Shanghai Chunan Corp., Shanghai, China) for meat products. The probe of the instrument was inserted into the meat sample for measurement and the value recorded. The arithmetic mean of the two measurements at the same point was taken as the result.

#### 2.2.2. The Tenderness of Meat

WBSF values were measured to evaluate texture of pork muscle. In this experiment, the sample was heated in a water bath at 80 °C, and when the central temperature of the sample reached 75 °C, the boiled sample was removed and cooled at 4 °C for 4 h. Three cuboids with cross-sectional area of 100 mm^2^ (10 mm × 10 mm) and length of not less than 30 mm were cut out from each cooked meat sample [19]. The long side of the cuboid was along the direction of muscle fibers. Then the meat cuboids were sheared perpendicular to the fiber direction by a testing machine (EZ-SX, Shimadzu Corp., Kyoto, Japan). The results from three independent measurements were averaged to yield the final index value.

#### 2.2.3. Myofibrillar Fragmentation Index

MFI was measured according to Culler et al. [20], whereby 4.0 g meat samples were added with 40 mL pre-cooled MF buffer (100 MMO/L KCl, 20 mmol/L P_3_O_4_, 1 mmol/L EDTA, 1 mmol/L MgCl_2_, 1 mmol/L NaN_3_, pH 7.0). The samples were homogenized at high speed three times (4 °C, homogenized for 20 s every 1 min), and the samples were centrifuged (4 °C 1000× *g*, 15 min). The supernatant was removed, the precipitate was suspended with 10 mL of precooled MF buffer, and connective tissue fragments were removed by filtering with 150 mesh filter cloth. The myofibril extract was obtained after the filtrate. The protein concentration of myofibril extract was determined by biuret method [21]. Then, MFI buffer was used to adjust the protein concentration of suspension to 0.5 mg/mL. MFI value was determined according to the absorbance at 540 mm and Formula (1).

MFI = OD_540nm_ × 200 (1)

#### 2.2.4. Measurement of Food Safety Indicators

TVB-N value was determined by semi-micro nitrogen determination method according to China National Standard GB/T 5009.44 (2003) [22]. The calculation method is presented in Formula (2).

(2) $$X=\frac{\left(V_{1}-V_{2}\right)\times c\times 14\times 100}{m\times 10/100}$$

where: *X* is the content of TVB-N in the sample (mg/100 g); *V*_1_ is the volume of hydrochloric acid consumed by the measured sample (mL); *V*_2_ is the volume of empty consumed hydrochloric acid (mL); *c* is the actual concentration of hydrochloric acid standard solution (mol/L); and *m* is the sample mass (g).

TVC was determined by plate counting method for food microbiological testing [12] according to China National Standard GB 4789.2-2022 [23]. TVC was calculated as shown in Formula (3).

(3) $$N=\frac{\sum C}{\left(n_{1}-n_{2}\right)\times d}$$

where: *N* is the total number of colonies; *C* is the sum of the number of suitable plate colonies; *n*_1_ is the number of low-dilution plate colonies; *n*_2_ is the number of high dilution plate colonies; and *d* is the dilution factor.

### 2.3. Meat Classification Based on Physical and Chemical Indicators

Based on the changes of these physical and chemical indexes, meat samples were divided into three types: rigor mortis, aging, and spoilage meat.

Rigor mortis is characterized by the accumulation of acidic substances and a sharp increase in meat hardness. Meat products with the lowest pH and highest WBSF values were classified as rigor mortis meat. Aging meat is characterized by an increase in MFI and a decrease in WBSF values. The tenderization rate of meat continues to increase until WBSF values become insignificantly different from the “basic hardness” of meat. Once the WBSF value was no longer significantly reduced, the meat was classified as aged. Spoilage meat, on the other hand, is measured based on the total number of colonies and volatile base nitrogen, which are useful indicators of meat freshness. Distinguishing spoilage meat from aging meatis critical. Once the TVB-N or TVC value of meat surpasses the acceptable standard (TVB-N > 20 mg/100 g or TVC > 7 Log CFU/g), it is directly identified as spoilage regardless of its rigor mortis or aging classification. Proper classification can help to prevent the sale of low-quality meat products, which can lead to adverse health consequences for consumers.

To differentiate between rigor mortis meat, aged meat, and spoiled meat, 30, 40, and 30 representative samples were carefully selected and labeled based on changes in specific physical and chemical indexes. Notably, data for approximately 80 samples were discarded as they were in a transitional period.

### 2.4. Spectra Extraction and Modeling

To obtain the typical spectrum of the sample, the background of the image should be removed from the hyperspectral image [24]. The threshold was determined by histogram, and then the mask was made by binarization. The region of interest (ROI) and background were segmented. More details can be found in the papers of Kamruzzaman et al. [25] and Barbin et al. [26]. The average spectral value in ROI was taken as the sample’s spectrum. Background segmentation and spectral extraction were performed using software ENVI4.8 (ITT Visual Information Solutions Inc., Rockville, MD, USA).

Support Vector Machine (SVM), as a supervised and non-linear learning method, is widely used in statistical classification and regression analysis. By using non-linear mapping, SVM can make low-dimensional, linear inseparable samples in the input space separable in a high-dimensional feature space. The performance of SVM is affected by kernel function, error penalty parameter ‘C’, and kernel parameter ‘σ’. In this experiment, a particle swarm optimization (PSO) algorithm was used to search for optimal ‘C’ and ‘σ’. PSO is a new evolutionary algorithm with high accuracy and fast convergence, making it suitable for solving practical problems. In this paper, the spectral model based on PSO-SVM was implemented in the Matlab2022a software.

### 2.5. Wavelet Transform Analysis of Spectral Data

The amount of data produced by the HSI system can pose challenges for efficient data transmission and computation. To address this, WT was utilized as a preprocessing technique for the HSI data. In spectral signal analysis, the choice of mother wavelet is a vital element affecting the efficacy of signal feature extraction. There are seven commonly used mother wavelets: Haar wavelet (‘haar’), Daubechies wavelet (“db”), discrete Meyer pseudo-wavelet (“dmey”), Coiflet wavelet (“coif2”), Symlet wavelet (“sym2”), biorthogonal wavelet (“bior1.5”), and reverse biorthogonal wavelet (“rbio1.1”). In this paper, the authors used various mother wavelets to decompose the original spectrum. The resulting analytic wavelet coefficients (cA and cD) were used as input variables for calibration models.

Core Code:

[cA1, cD1] = dwt(signal, ‘wavename’) % The spectral signal is decomposed for the first time. wavename should be haar, db, dmey, etc.

[cA2, cD2] = dwt(cA1, ‘wavename’) % Step-by-step decomposition of cA coefficients, cA and cD of the next layer can be obtained.

## 3. Results

### 3.1. Physicochemical Changes of Pork during Postmortem Storage

**Figure 2:**
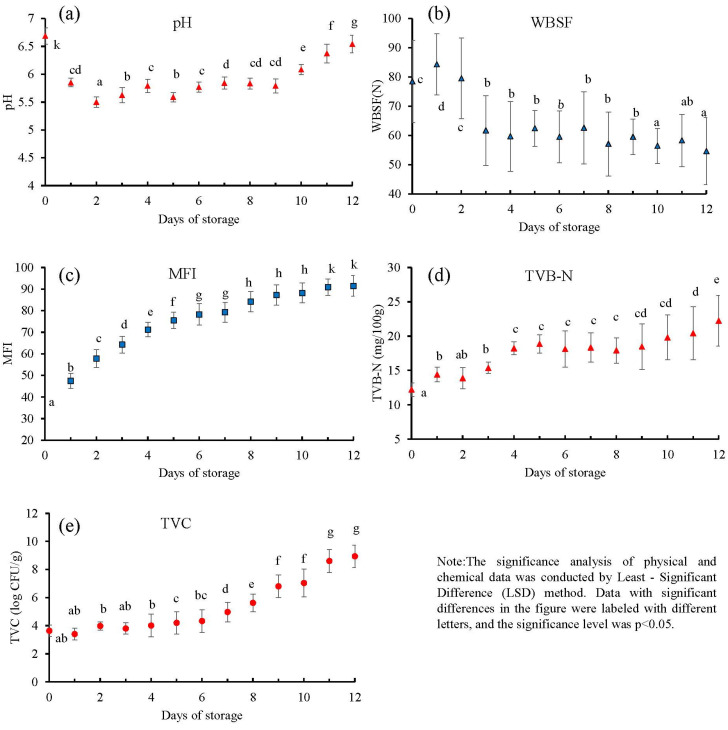
The pH value, WBSF, MFI, TVB-N, and TVC of pork during postmortem storage. (**a**–**e**) were the pH value, WBSF, MFI, TVB-N, and TVC of pork during postmortem storage respectively.

Figure 2 illustrates the multifaceted changes in pork during storage after slaughter. The pH of most pork samples significantly decreased during the first two days post-slaughter. Glucose can be converted into lactic acid in a hypoxic environment, while producing a small amount of ATP. In the subsequent stage, irreversible binding occurs between myosin and actin in the muscles, causing permanent muscle contraction, i.e., rigor mortis. As a result, the WBSF value increases and its water-holding capacity diminishes within two days of slaughter, marking the transition from hot fresh meat to the rigor mortis period [27,28,29]. Figure 2 further demonstrates that the pH of postmortem pork rises as storage time increases, which is largely due to muscle acidity discharge. However, in the later stages of storage, the pH increases rapidly as meat spoilage takes hold, likely due to the release of biogenic amines from meat decomposition.

Throughout cold storage, the MFI increases and the WBSF value decreases over time. This phenomenon is primarily attributed to the action of calpain enzymes, which catalyze the degradation of cytoskeletal proteins within the muscle fibers, leading to their structural breakdown and gradual tenderization of the meat. Additionally, it is worth noting that alongside calpains, cathepsins—a class of lysosomal proteases known to play crucial roles in proteolytic processes during physiological conditions—also contribute to the proteolysis of muscle proteins. Cathepsins, particularly active under acidic conditions typically found in lysosomes, can further enhance the tenderization process by degrading various protein substrates within the muscle tissue.

Although the shear force value exhibits a rapid decline in the initial phase of storage, it stabilizes in the subsequent stages. This stabilization reflects the influence of factors such as muscle fiber thickness and collagen content on the fundamental texture of meat products. Moreover, water evaporation from the meat surface during storage contributes to increased dryness and hardness. As a result, the hardness of pork approaches a plateau where it no longer decreases substantially, indicating that the combined effects of calpains, cathepsins, and physical changes converge to determine the ultimate tenderness and texture of the stored meat.

The reproduction of spoilage microorganisms is a major contributing factor to the poor sensory traits and quality of meat. Common microorganisms that cause spoilage in meat include *Pseudomonas*, *Clostridium*, *Micrococcus*, *Alcaligenes*, and *Lactobacillus*. When microorganisms reproduce on a large scale, they destroy the nutritional value of meat and cause discoloration. In addition, pathogens and toxins may be formed, which pose potential harm to human health. Throughout the storage period, TVC and TVB-N values gradually increased, with some samples exceeding the food safety standard during the late period. Although the total number of colonies briefly decreased in the first two days of cold storage, the values gradually rose thereafter.

### 3.2. Spectral Characteristics of Meat in Different Postmortem Periods

**Figure 3:**
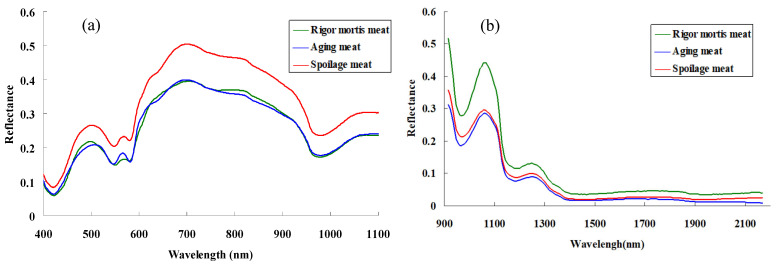
The average spectra of pork meat at different storage periods. (**a**) was the spectra of the samples in in the visible light to short wave near-infrared range (400–1000 nm), and (**b**) was the spectra of the samples in the near-infrared range (1000–2200 nm).

The average spectral curves of meat in different periods are shown in Figure 3. In the early and middle stages of cold storage, the spectral values of pork in the visible band were very close. As the refrigerating time was extended, the spectral curves of pork had many changes in detail. The average spectral value of spoiled meat stored for a long time was generally higher than that of non-spoiled meat. The reason for the increase of spectral value was related to the loss of heme, oxidation, and color change of meat during storage. In addition, another important reason was that moisture has a strong absorption to the spectrum, and moisture evaporation in meat storage reduces the absorption to the spectrum.

In the near infrared bands, the spectral value was higher in the early stage, and decreased after a period of refrigeration. The spectrum of rotten meat could not be easily distinguished from that of non-rotten tender meat. Generally, there were only two spectral peaks in the near infrared band. The spectrum after 1400 nm was basically a straight line, and the characteristic amount of the spectrum was less in the near infrared than in the visible band. Therefore, it was more suitable to analyze the aging progress of meat by visible light spectroscopy.

### 3.3. Wavelet Transform Analysis of Meat Spectrum

Data compression techniques are often applied in HSI data analysis. However, there was no significant difference in spectral curve between rigor and aged meat. The traditional method of selecting characteristic bands for modeling may lose key information and affect model performance. A possible solution is to use wavelet transform, which operates by layering spectral morphological information while preserving the original data. This method is better suited to identify the characteristic values of spectral curves. In this study, seven different mother wavelets, Haar wavelet, Daubechies wavelet, discrete Meyer pseudo-wavelet, Coiflet wavelet, Symlet wavelet, biorthogonal wavelet, and reverse biorthogonal wavelet, were used to transform pork spectra. The results showed that Daubechies 5 (‘db5’) as mother wavelet could extract feature information from hyperspectral images well.

**Figure 4:**
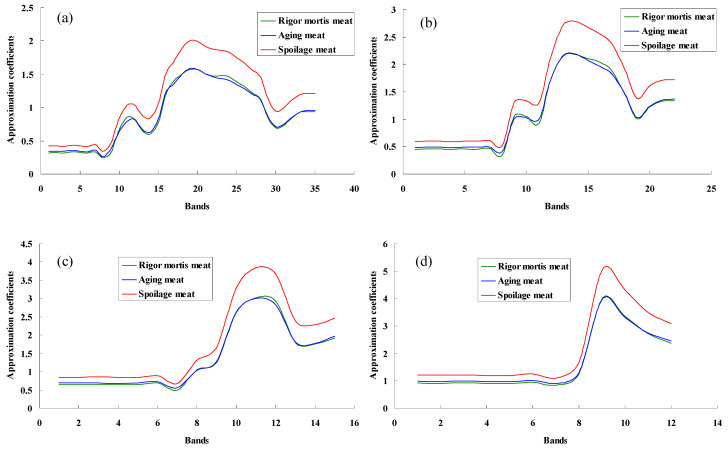
Wavelet approximation coefficients obtained from pork spectra treated with “db5”. (**a**–**d**) were the fourth-, fifth-, sixth-, and seventh-order wavelet approximation coefficients, respectively.

Figure 4 shows the cA obtained by ‘db5’ wavelet transform. The cA of the wavelet could describe the low frequency information of the original signal, that is, the shape of the pork spectrum. After several wavelet transformations, many details of the infrared spectra curve were stripped out. The spectral curve became smoother because many small peaks decreased. Meanwhile, there was no significant change in the contour of the spectra, which was consistent with the original spectra.

**Figure 5:**
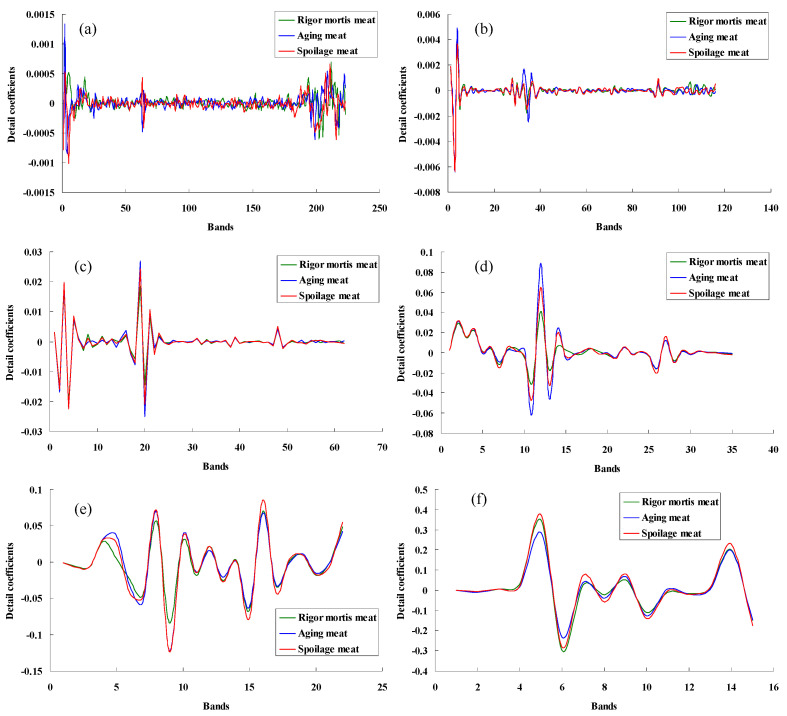
Wavelet detail coefficients of pork meat using “db5”. (**a**), (**b**), (**c**), (**d**), (**e**), and (**f**), respectively, were the first, second, third, fourth, fifth, and sixth wavelet detail coefficients.

The cDs of WT describe the high frequency information of the original signal, that is, the detailed information of the spectra. Upon analysis of the wavelet detail coefficients depicted in Figure 5, the first and second cDs exhibited more noise. The fourth and fifth wavelet detail coefficients highlighted the significant difference in spectral details. Notably, the disparity between the rigid and aged meat became more pronounced after the wavelet transform relative to the original spectrum. Additionally, the spectral data’s dimensions reduced significantly. The original spectrum (400–1100 nm) consisted of 438 bands, while the number of variables from cD1 to cD7, after decomposition, reduced to 223, 116, 62, 35, 22, 15, and 12, respectively. The experimental results demonstrated the wavelet transform’s three distinct functions: denoising, data compression, and feature extraction.

### 3.4. Evaluating the Aging Progress with SVM Model

The SVM algorithm is known for its exceptional ability in non-linear classification. However, when dealing with input data with many dimensions, the computation and modeling time can become overwhelming. In our experiment, using 438 original spectral bands as input variables resulted in computer memory overflow and inability to calculate.

To combat this issue, the cA and cD of WT in different layers were taken as input variables of the SVM model. This significantly reduced the operation intensity. Our study was conducted with 100 samples randomly divided into 66 calibration sets and 34 verification sets. The classification accuracy of the model under various orders was recorded in Table 1. By decomposing the wavelet transform layer by layer, the time required for input dimension and training model was considerably decreased. This led to an improvement in the efficiency and accuracy of classification. The model based on cA4 and cD4 coefficients had the highest classification accuracy. The training set had a 100% accuracy rate, while the verification set had an accuracy rate of 97.06%. This was due to the fourth-order decomposition’s ability to preserve the spectral morphology and extract intricate features. Our findings demonstrate that hyperspectral imaging technology accurately identifies different aging stages of pork samples.

### 3.5. Display of Postmortem Aging Progress with Color Image

**Figure 6:**
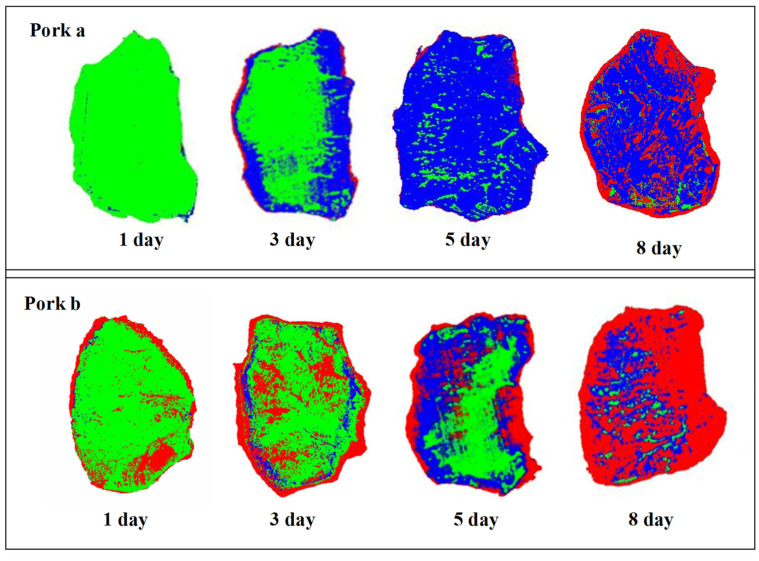
Mapping the aging process of different pork meat. The green area represented the “Rigor Mortis Phase”, the blue area signified the “Aging Phase”, and the red area indicated the “Spoilage Stage” in meat processing timelines.

By substituting the spectral value of all the pixels in the meat sample into the spectral model, a meat aging distribution map could be estimated [30]. The technology also allows for the dynamic evaluation of pork meat’s daily variation through the calculation and visualization of spectral images of pork samples. The analysis revealed that the aging of meat occurs initially at the edges of the meat and progressively increases with time. The onset of microbial deterioration also begins at the edges of the meat. Additionally, each pork sample ages at different rates and completion times, which are clearly depicted in Figure 6.

The research showed that Pork a had aged perfectly after five days of storage, with ideal hygienic conditions and slow spoilage rate, resulting in high-quality sales. Pork b, on the other hand, still had rigor mortis areas after fiven days of storage, and its spoilage rate was quick, indicating that the refrigeration environment needed improvement [31]. This technology helps meat producers monitor the aging progress of each piece of meat and determine whether it is suitable for storage or should be removed from storage, ultimately ensuring high-quality meat products.

## 4. Discussion

This study delves into a series of experiments to explore the physicochemical changes in pork during postmortem storage and their impact on meat quality. Initially, it was observed that the pH value of pork significantly decreases shortly after slaughter, followed by a gradual increase. This process is associated with the discharge of muscle acidity and the release of biogenic amines during meat spoilage. Simultaneously, as the cold storage time increases, the tenderness of the meat gradually improves, which is related to the destruction of muscle fiber structure and changes in collagen content. Furthermore, the growth in microbial reproduction has a significant influence on meat quality, especially in the later stages of storage, where the increase in total colony count and TVB-N values indicate that the meat has exceeded food safety standards.

In terms of spectral characteristics, hyperspectral imaging technology was used to analyze pork samples at different postmortem periods. It was found that as storage time extended, the spectral curves of the meat underwent significant changes in detail, mainly due to the loss and oxidation of hemoglobin and color variations. Further, wavelet transformation analysis was applied to the spectral data of the meat. By selecting appropriate wavelet basis functions, characteristic information was successfully extracted, achieving noise reduction and data compression. This provided critical information for subsequent SVM model classification.

In assessing the aging process of meat, an SVM model based on wavelet transform coefficients was constructed. The model improved classification accuracy and efficiency while reducing computational complexity. Experimental results show that the model can accurately identify pork samples at different aging stages, providing an effective monitoring tool for meat producers.

Lastly, the aging process of pork was illustrated through color images, revealing differences in the aging rate and completion time among different pork samples. This has important implications for guiding storage decisions in actual production. In summary, this research not only deepens our scientific understanding of postmortem pork changes but also provides the meat industry with a practical set of quality control methods. Future work will further optimize the model to improve prediction accuracy and apply this technology to actual production for more intelligent and automated meat quality inspection and management.

## Figures and Tables

**Table 1:** Classification accuracy for different pork meat using SVM models.

DWT Decomposition	Dimension	Classification Accuracy (%)	
		Training Set	Prediction Set
Original spectra	438	-	-
1-Level	223 + 223	-	-
2-level	116 + 116	96.97	93.94
3-level	62 + 62	98.48	94.12
4-level	35 + 35	100	97.06
5-level	22 + 22	98.48	93.94
6-level	15 + 15	95.45	91.18
7-level	12 + 12	93.93	88.24

## Data Availability

The original contributions presented in the study are included in the article, further inquiries can be directed to the corresponding author.
